# 

**Published:** 1891-02-07

**Authors:** 


					imnwKNtv^ mammwm
r INFANTS 42 and INVALIDS.
n
No. 228. YoL IX".~i Saturday,
Febrttary 7th, 1891.
[Onjs Pen.vy.

				

